# Impact of Enzymatic Degradation on the Material Properties of Poly(Ethylene Terephthalate)

**DOI:** 10.3390/polym13223885

**Published:** 2021-11-10

**Authors:** Teresa Menzel, Sebastian Weigert, Andreas Gagsteiger, Yannik Eich, Sebastian Sittl, Georg Papastavrou, Holger Ruckdäschel, Volker Altstädt, Birte Höcker

**Affiliations:** 1 Department of Polymer Engineering, University of Bayreuth, Universitätsstraße 30, 95447 Bayreuth, Germany; Teresa.Menzel@uni-bayreuth.de (T.M.); bt716629@uni-bayreuth.de (Y.E.); holger.ruckdaeschel@uni-bayreuth.de (H.R.); volker.altstaedt@uni-bayreuth.de (V.A.); 2 Department of Biochemistry, University of Bayreuth, Universitätsstraße 30, 95447 Bayreuth, Germany; Sebastian.Weigert@uni-bayreuth.de (S.W.); Andreas.Gagsteiger@uni-bayreuth.de (A.G.); 3 Department of Physical Chemistry II, University of Bayreuth, Universitätstraße 30, 95447 Bayreuth, Germany; sebastian.sittl@uni-bayreuth.de (S.S.); Georg.Papastavrou@uni-bayreuth.de (G.P.)

**Keywords:** polymer degradation, microplastic, nanoplastic, PETase, crack formation, fatigue crack propagation resistance, BHET, enzymatic degradation, enzyme, *Ideonella sakaiensis*, bis(hydroxyethyl)terephthalate

## Abstract

With macroscopic litter and its degradation into secondary microplastic as a major source of environmental pollution, one key challenge is understanding the pathways from macro- to microplastic by abiotic and biotic environmental impact. So far, little is known about the impact of biota on material properties. This study focuses on recycled, bottle-grade poly(ethylene terephthalate) (r-PET) and the degrading enzyme PETase from *Ideonella sakaiensis*. Compact tension (CT) specimens were incubated in an enzymatic solution and thermally and mechanically characterized. A time-dependent study up to 96 h revealed the formation of steadily growing colloidal structures. After 96 h incubation, high amounts of BHET dimer were found in a near-surface layer, affecting crack propagation and leading to faster material failure. The results of this pilot study show that enzymatic activity accelerates embrittlement and favors fragmentation. We conclude that PET-degrading enzymes must be viewed as a potentially relevant acceleration factor in macroplastic degradation.

## 1. Introduction

Since the discovery of microplastics (MPs) in 2004 [1], particles have been detected in almost every natural environment. Primary MP is already produced on a micrometer scale, whereas secondary MP arises by degradation and fragmentation of macroplastic. As the amount of secondary MP to be found in nature is drastically larger than that of primary MP, the degradation of macroplastic has recently gained new attention [2,3,4]. Once in the environment, polymers are exposed to a range of external environmental impacts. These can be categorized as abiotic factors such as UV-radiation, temperature, humidity, and mechanical stress, and biotic factors such as living or dead organisms, e.g., biofilm formation by bacteria, fungi, algae, or ingestion [5,6]. A complex interplay of natural stress factors is supposed to lead to molecular degradation, fragmentation, and therefore MP formation [7,8,9]. However, knowledge of the underlying processes is still lacking. It is evident that material properties decisively influence the fragmentation towards MP and dramatically change during this process, creating a feedback loop on the degradation itself.

Regarding biotic degradation, polyesters, especially polyethylene terephthalate (PET), are the subject of many studies due to their molecular structure [10]. Ester bonds are omnipresent in key positions within metabolic networks and biological molecules. Although other common polymer bond types like C-C are more challenging in a biological perspective, a whole variety of hydrolases is known to deal with ester bonds in nature. Particularly in recent years, PET-degrading enzymes have come into focus as a new perspective for biological MP decomposition and recycling applications [11]. In this regard, cutinase enzymes are of particular interest, as their aliphatic ester substrate, cutin, has a chemical resemblance to PET. Consequently, all PET-degrading enzymes with relevant activity known to date can be assigned to this group of cutinases [10], including the best-studied variants TfCut-1/2 (*Thermobifida fusca*) [11], LCC (uncultured organism) [12], and PETase (*Ideonella sakaiensis*) [13]. The latter piqued researchers’ interest, as it appeared as the first evolved PET-degrading enzyme with substantial activity at ambient temperatures. This activity profile, combined with the presence of potential PET-degrading enzymes in different ecosystems [14], suggests the altering of PET’s material properties upon biological activity under real conditions. 

On a chemical level, abiotic factors are relatively well characterized: Photooxidation by UV radiation and oxygen leads to radical-induced chain scission and the formation of new polar functional end groups, e.g., carboxylic acids, aldehydes, hydroxides, or peroxides [15,16,17,18]. Hydrolytic degradation strongly depends on an interplay of humidity, temperature, pH, and the crystallinity of the material [17,19,20,21]. Both processes lead to a decrease in the molecular weight. Regarding material properties, it is well known that the transition of the molecular weight below the critical molar mass (M_c_, 17 kg/mol for PET [22]) causes a change in material behavior from ductile to brittle [23]. In general, abiotic degradation and stepwise embrittlement accelerate the fragmentation upon external mechanical forces like wind and waves [24,25]. However, except for one study [22], there is a lack of detailed information about changes in mechanical properties on PET depending on environmental impact in the literature. When it comes to biotic degradation of PET and its impact on the material, even less is known besides the investigation of changes in PET crystallinity during enzymatic degradation [26]. 

A sensitive method for the determination of micromechanical material changes can be supplied by mechanical testing under dynamic load [27,28]. This technique gives precise information on the fatigue crack propagation (FCP) behavior at various crack propagation speeds and is claimed to be the most sensitive regarding the relationships between the polymer structure and deformation mechanism. The stress state at the crack tip is well defined compared to conventional tensile or impact testing. This allows possible correlations between the interaction of the crack tip, propagation of the fatigue crack, and the sensitivity of a specific polymer to environmental stress. It is well known that the FCP rate is strongly affected by the degree of crystallinity and the tie molecule density of polymers [29,30], as well as by their molecular weight [31]. Eventually, the linear dependency of the FCP rate on the applied stress intensity, indicating stable crack growth, provides qualitative information about improved or deteriorated material behavior [32].

To the best of our knowledge, there have been no studies focusing on the impact of biotic stresses on macroscopic PET properties. For detailed information on the formation of MP on their pathway from macro to micro, biotic factors on material properties must be considered. Within this feasibility study, we combine the methods and techniques of biochemistry and engineering sciences. This interdisciplinary combination allows first insights into the change of material properties in a laboratory-controlled biotic degradation process. Overall, the aim of our study is to understand the impact of biotic degradation by PETase on a macro- and microscopic level by focusing on PET material properties. Insights into the underlying processes enable us to comprehend how biotic degradation impacts PET fragmentation.

## 2. Materials and Methods

A commercially available, recycled, low-molecular-weight PET (CleanPET^®^ FK) (Veolia Deutschland GmbH, Berlin, Germany) was used with a number-averaged molecular weight of M_n_ = 30.155 g/mol and dispersity Ð = 1.8 determined by GPC measurements (Agilent 1200 Series, Agilent Technologies, Waldbronn, Germany) with HFIP with potassium trifluoroacetate (4.8 g in 600 mL HFIP) as solvent. The PET flakes were acquired from disposable, post-consumer bottles and therefore contained an undeterminable amount of several additives. Thus, the material composition reflected a realistic condition regarding real-life environmental conditions. 

### 2.1. Protein Production and Purification

The gene of *is*PETase was cloned via Gibson assembly [33] in a pMAL-p4x vector, in which the MPB sequence was replaced with the mauC signal peptide for periplasmic expression. *E. coli* BL21 cells containing the plasmid were grown in TB media at 37 °C; after an OD600 of 1.5 was reached the temperature was lowered to 18 °C and protein expression was induced with a final concentration of 300 µM IPTG. After 18 h, cells were harvested and resuspended for sonication in lysis buffer (300 mM NaCl, 40 mM imidazole, 50 mM phosphate pH 7.4). The lysate was clarified through centrifugation (50,000× *g*) and vacuum filtration (0.2 µm filter), and subsequently loaded onto a NiIMAC column (HisTrap FF 5 mL, Cytiva Europe, Freiburg, Germany). After loading and washing, the protein was recovered from the column with elution buffer (300 mM NaCl, 400 mM imidazole, 50 mM phosphate pH 7.4). For final polishing, the protein was applied to a SEC column (Superdex 75pg 26/60, Cytiva) equilibrated with SEC buffer (150 mM NaCl, 25 mM HEPES, pH 7.4). A total of 100 µL aliquots of the enzyme with a concentration of 25 µM were flash frozen and kept at −80 °C until further use.

### 2.2. PET Sample Preparation 

Compact tension (CT) specimens with a width and thickness of 40 and 4 mm, respectively, were prepared by injection molding (Arburg Allrounder 470H 1000-170, Arburg GmbH, Loßburg, Germany). Each sample was tapped with a new razor blade into the V-notch to create a sharp crack. For a time-dependent degradation study of the surfaces, squares with 2 cm edge length were sawn out of the injection-molded CT specimen for easier handling. 

### 2.3. Sample Incubation with PETase

For the time-dependent degradation study, the squares with 2 cm edge length were fully covered with an enzyme-buffer solution in a 50 mL centrifuge tube. The enzyme concentration was constantly set to 200 nM in a reaction buffer of 50 nM NaCl and 50 mM borate at pH 8.5. A control sample was covered under the absence of enzyme with a buffer solution only. All samples were incubated at 30 °C for 24 h, 48 h, and 96 h, respectively. The sample without enzyme is further referred to as control. The PET samples, additionally incubated with enzyme, are further referred to as PET*24 h, PET*48 h, and PET*96 h.

**Figure 1:**
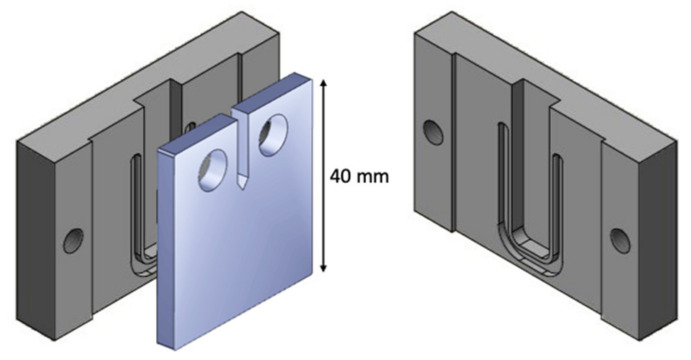
CT specimen and sample holders for the incubation with enzyme-buffer solution. The CT specimen is placed between two perfectly fitting parts, and tightly secured with screws. The indentations are filled either with buffer or enzyme-buffer solution. To avoid leakage of the solutions, U-shaped seals are fixed between specimen and sample holder. The setup ensures the total coverage of the specimen with solution within the expected crack propagation direction.

For mechanical testing, the CT specimens were placed in purpose-built holders made of stainless steel (Figure 1). This setup ensured the sufficient coverage of solution at the notch and along the expected crack propagation. The sample holder was assembled with a specimen and filled with 2 mL of the abovementioned enzyme-buffer solution. Again, a specimen incubated with buffer solution only serves as control sample. The sample holders were placed in a gastight container comprising additional reaction buffer on the bottom to minimize evaporation of the solution within the sample holder. The specimens for mechanical testing were incubated at 30 °C for 96 h. After exposure, the CT specimen was rinsed with water and a standard PET drying procedure (6 h at 140 °C) was applied to eliminate the influence of moisture in further characterization steps.

For this study, incubation parameters such as buffer composition, temperature, and enzyme concentration were chosen primarily to optimize enzymatic activity. Although they did not necessarily reflect conditions in natural settings, they allowed for the best results to establish the methodology. 

### 2.4. Ultra-High Performance Liquid Chromatography (UHPLC)

The water-soluble degradation products of PET were quantified with a Thermofisher RS3000 UHPLC system equipped with a Phenomenx Kinetex 1.7 µm EVO C18 (100 Å, 50 mm × 2.1 mm) reversed-phase column. For sample preparation, one part of the sample buffer solution of the PET control, PET*24 h, PET*48 h, and PET*96 h after incubation was mixed with four parts acidic acetonitrile (1% formic acid) and centrifuged at 21,000× *g* for 10 min. A quantity of 1 µL of the supernatant was applied to the column running a gradient from 100% solvent A (water + 0.1% trifluoroacetic acid) to 20% solvent A and 80% solvent B (acetonitrile) at a flow rate of 1.3 mL/min and the column was heated to 55 °C. The product amounts for TPA, MHET, and BHET were quantified based on calibration runs. For the time-dependent monitoring of the enzymatic activity, the three degradation products were summed up to the total product concentration for better comparison. 

### 2.5. Optical and Topographical Characterization

To visualize the enzymatic degradation on a microscopic scale, field-emission scanning electron microscopy (FESEM) was performed on the surfaces with a Zeiss Ultra plus (Carl Zeiss Microscopy Deutschland GmbH, Oberkochen, Germany) at an acceleration voltage of 3 kV for the control and the enzyme-degraded samples. All samples were sputtered with 1.3 nm platin at a Cressington Platin-Sputter Coater 208HR (TESCAN GmbH, Dortmund, Germany) and additionally steamed with 20 nm carbon at a Leica EM ACE 600 (Leica Microsystems GmbH, Wetzlar, Germany). 

The surface topography of the enzymatically degraded PET*96 h and control PET sample was acquired by atomic force microscopy (AFM) imaging in PeakForce tapping mode in air. All images were acquired using a Dimension Icon AFM (Bruker Corporation Billerica, Massachusetts, USA) equipped with a NanoScope V controller. For imaging, ScanAsyst Air cantilevers (Bruker Nano Inc., nominal spring constant 0.4 N/m, nominal resonance frequency 70 kHz) were used. The PeakForce frequency was set to 2 kHz with an amplitude of 150 nm. The AFM images were processed with NanoScope Analysis software (version 1.80, Bruker Nano Inc.). In an additional set of experiments, the samples, i.e., enzyme-degraded and control sample surfaces, were treated for about 30 s by a CO_2_ gun (SnowJet, Tectra GmbH, Frankfurt, Germany) in order to remove potential organic contaminants.

### 2.6. Thermal Characterization

Differential scanning calorimetry (DSC) was examined with a Mettler Toledo DSC I (Mettler-Toldeo GmbH, Gießen, Germany) with 8–10 mg sample material. Material was collected between 0 and 1 mm depth from the surface. The samples were heated under an N_2_ atmosphere from −10 °C to 300 °C (1st heating run), and cooled to −10 °C again after an isothermal stage of 5 min at 300 °C. For determination of the degree of crystallinity x_c_ a fusion enthalpy of ΔHm° = 140.1 J/g for a hypothetically 100% crystalline PET was used, based on the literature [34].

### 2.7. Mechanical Characterization

The FCP behavior was determined according to test method ISO 15850/ASTM E647 at 23 °C and a relative humidity of 50% on a servo-hydraulic testing machine (IST IPLH10I, Schenck, Germany) applying a dynamical load with a frequency of 10 Hz to the samples. The stress intensity factor’s ΔK = K_max_ − K_min_ amplitude was increased in proportion to the crack length with a constant R = K_min_/K_max_ of 0.1. The crack was supposed to grow perpendicular to the load within the solution covered area until the end of the sample. For measurement of the crack opening displacement during crack growth, a clip-on extensometer (632.29-30, MTS Sensor Technology GmbH & Co. KG, Rottenburg am Neckar, Germany) was used. Each experiment loaded under tension mode was repeated at least three times. Analysis was done with the software R [35]; Zone II was defined manually between ΔK = 2.2 and ΔK = 3.05.

## 3. Results and Discussion

### 3.1. Analysis of Soluble Products

**Figure 2:**
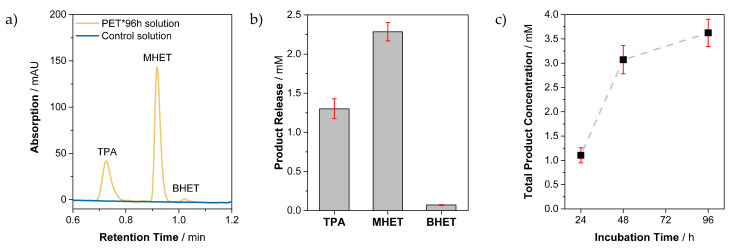
Analysis of soluble products. (**a**) UHPLC results of the control buffer solution (blue) and enzyme buffer solution (orange) of PET*96 h. For the control buffer solution, no release products could be detected. (**b**) Product concentrations of PET*96 h to display the product composition in mM and (**c**) total product concentration of PET*24 h, PET*48 h, and PET*96 h solution versus incubation time.

To monitor enzymatic degradation on the molecular level, the control buffer solution and enzyme buffer solution were analyzed by ultra-high-performance liquid chromatography (UHPLC) after the given incubation time of 24 h, 48 h, and 96 h. Regarding product composition, the exemplarily plotted buffer solution of PET*96 h contained the typical degradation products terephthalic acid (TPA), mono-(2-hydroxyethyl)terephthalic acid (MHET), and bis(hydroxyethyl)terephthalate (BHET) (Figure 2a). In the case of the control buffer, none of the typical degradation products could be detected (Figure 2a). The quantification of the degradation products for PET*96 h (Figure 2b) gave high amounts of MHET and TPA with 2.3 mM and 1.3 mM, respectively. Further, small amounts of 0.1 mM BHET were identified. For the time-dependent study, the total product concentration for PET*24 h, PET*48 h, and PET*96 h was determined. The total product concentration increased in relation to the incubation time, with a slight slowdown after 48 h (Figure 2c). The results of the solution analysis represent a typical activity profile for PETase on PET with MHET as the dominant product [13].

The UHPLC measurements verify that the observed PET degradation could only be attributed to enzymatic activity and no side reaction or autohydrolysis occurred during incubation. Furthermore, the results reflect the consistency in our setup, including sample generation and incubation. True to expectations [36], the total product release, consisting of TPA, MHET, and BHET, steadily rose with increasing exposure time (Figure 2c). This indicates a constantly ongoing degradation of PET by PETase over incubation time. In summary, the setup ensured a reliable procedure for monitoring enzymatic activity of a macroscopic substrate on the molecular level. 

### 3.2. Characterization of PET Material Properties

**Figure 3:**
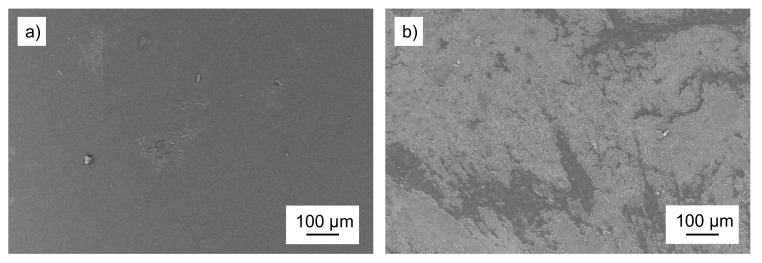
SEM micrographs for comparison of the (**a**) control PET surface and (**b**) PET surface of PET*96 h after incubation.

To investigate the result of degradation on a visible level, scanning electron microscopy (SEM) micrographs of the surfaces of the control and incubated PET samples were recorded (Figure 3).

In the case of the control PET, the micrographs displayed a smooth and unaffected surface (Figure 3a). The exposure of the samples to PETase enzyme led to visible degradation by surface erosion (Figure 3b), indicating successful trials and degradation. The overview at low magnification depicted the spatial heterogeneity of the enzymatic treatment, reflected by the presence of unaffected and degraded areas. Furthermore, the investigation of the control sample surface verified that it was not affected by autohydrolysis of the buffer solution and degradation only arose due to the impact of enzymes. For a deeper investigation of the topographical surfaces’ changes, a time-dependent surface SEM study was performed. 

**Figure 4:**
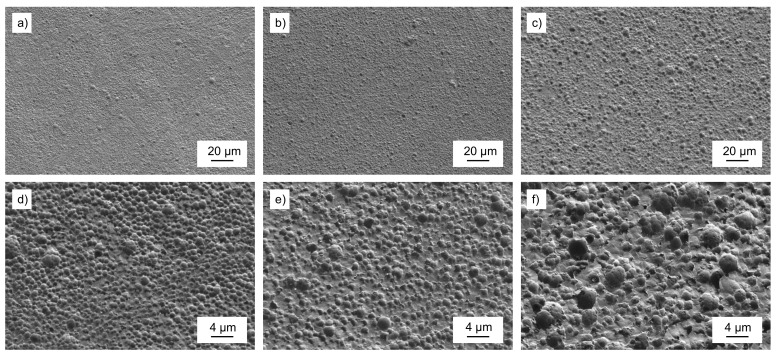
Time-dependent SEM micrographs at different degradation stages of (**a**) PET*24 h, (**b**) PET*48 h, and (**c**) PET*96 h and with higher magnification at (**d**) PET*24 h, (**e**) PET*48 h, and (**f**) PET*96 h.

The SEM micrographs of the time-dependent degradation study are shown in Figure 4. The affected areas at higher magnitudes showed the presence of colloidal structures upon enzymatic treatment. They constantly grew with increasing incubation time to a diameter of approx. 2 µm. However, at that point we could not distinguish between surface erosion and the congregation of side products on the PET surface during the enzymatic degradation. 

**Figure 5:**
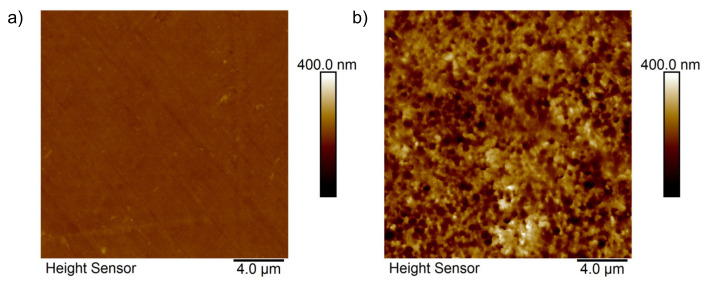
PeakForce Tapping mode AFM images of the control PET surface (**a**) and PET*96 h surface (**b**).

To corroborate the topography changes upon enzymatic treatment as determined by SEM, we studied the PET surface of the control and enzyme-treated PET*96 h sample additionally by AFM. For those measurements we used PeakForce Tapping mode (Figure 5) as the imaging mode to correlate the colloidal growth with surface roughness. 

The blank PET surface was smooth (Figure 5a). The sample showed a homogeneous surface without any distinct topographical features. On the other hand, the PET*96 h surface, which was incubated and treated with the PETase, showed a significant increase in roughness. Figure 5b bears distinct differences in surface topography with features that are absent for the bare control PET substrate (cf. Figure 5a). The observed increase in surface roughness is in line with the findings from the SEM investigations (cf. Figure 4) and indicates that the enzymatic treatment was accompanied by changes in the surface topography. In order to further quantify this finding, the arithmetic surface roughness was evaluated for at least three AFM images for each type of PET sample. The untreated surface bore a roughness of 7.3 nm ± 3.7 nm and the PETase-treated samples bore a roughness of 37.0 nm ± 7.8 nm, respectively.

**Figure 6:**
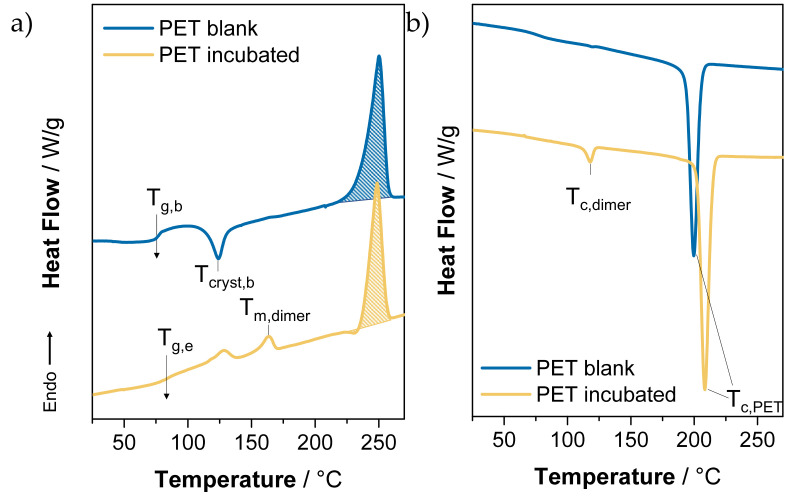
DSC thermograms of the first heating run (**a**) and first cooling run (**b**) for PET blank (blue) and PET incubated with PETase (orange). For the enzyme-treated samples, the rise of a second melting peak was identified as BHET dimer.

Differential scanning calorimetry (DSC) measurements allow inferences to be drawn from thermal transition temperatures about molecular arrangements. Until now, the focus in literature dealing with enzymatic degradation has been the identification of degradation products in solution [13,26,37], but no concrete measurements have been carried out on PET sample surfaces. DSC thermograms of the heating curves and cooling curves for the PET control and PET*96 h sample are shown in Figure 6. The DSC samples were taken along the crack of a CT specimen at a 0–1 mm sample depth.

In the first heating curve (Figure 6a), both samples showed the PET typical melting point T_m_ = 250 °C [38]. Beside the glass transition T_g,b_ = 76 °C, the control sample additionally showed an exothermic peak at T_cryst,b_ = 120 °C related to amorphous phase crystallization. In the case of the incubated PET*96 h sample, the glass transition showed a weak signal at T_g,e_ = 84 °C. The increase in the glass transition temperature of incubated PET can be explained by the emergence of crystalline units of two different molecular species in a range from 109 to 172 °C (Figure 6a). They were supposed to immobilize the amorphous chains [39], which led to the slight increase of 8 K. 

The appearance of these new melting peaks can be attributed in detail to PET oligomers with a varying length of one and two monomer repeating units, respectively [40]. Whereas the weaker signal from 109 °C to 135 °C could be attributed to monomeric BHET, the melting point at T_m,dimer_ = 164 °C clearly indicates the presence of BHET dimer [41,42,43]. The observed BHET dimer was water-insoluble and therefore not detectable in UHPLC measurements of the solutions. We conclude that the observed colloidal structures (Figure 4) on the surface were composed of BHET oligomer species. However, the accumulation of BHET and its dimer indicates that they were not preferably degraded by the PETase. 

A possible explanation for this behavior lies in the topology of the enzyme itself. Although there is no experimental proof for the exact binding mode of PET substrate to PETase, it is known that the PET binding site includes an L-shaped shallow grove neighboring the active site. Several studies using computational calculations to dock a PET oligomer in the active and binding site yielded a binding pose that provides space for the equivalent of either a BHET trimer or tetramer [26,44,45]. Shorter substrates such as the BHET dimer are not able to cover the full binding site, leading to a potentially decreased number of atomic contacts that contribute to the binding of the substrate. This would result in a higher affinity for higher oligomers in enzymatic catalysis followed by an accumulation of BHET dimer. Consequently, enzymatic activity might be underestimated when only soluble products are tracked. 

The degree of crystallinity decreased with enzymatic treatment from x_c,b_ = 22% for the PET control to x_c,e_ = 18% for the PET*96 h sample. It is known that high crystallinity impairs enzymatic activity, which led to the conclusion that PETase was mainly active in the amorphous regions. If this was the case, crystallinity should have increased. However, our results show that the degree of PET crystallinity decreased after enzymatic incubation, as reported in previous studies [22]. We assume that this lower degree in crystallinity of PET was not directly caused by enzymatic attack on crystalline regions, but rather by BHET species disrupting the crystalline order on the surface, explaining how crystallinity can decrease despite preferred enzymatic degradation of amorphous regions. The presence of BHET was also observed in the cooling curves (Figure 6b). Whereas the control sample only showed one sharp recrystallization peak at T_c,PET_ = 200 °C typical for PET, the PET*96 h cooling curve showed an additional peak at T_c,dimer_ = 117 °C, corresponding to BHET dimer recrystallization. The earlier onset of PET incubated with PETase recrystallization could be a nucleation effect induced by the additional oligomeric fraction.

**Figure 7:**
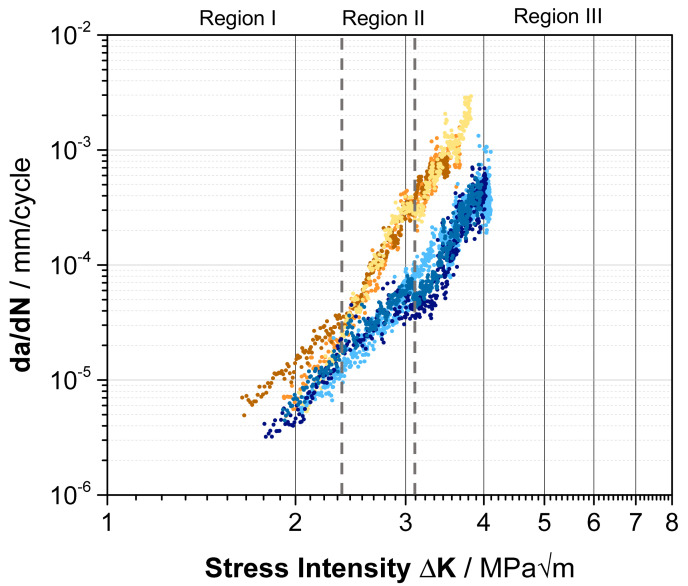
FCP behavior of the PET control (light blue, blue, dark blue) and PET*96 h (yellow, orange, brown) samples in a double logarithmic scale with the division into three relevant regions, marked by the dashed lines and grey color.

To further investigate the impact of enzymatic degradation on the surface and therefore on the material’s properties, dynamic mechanical measurement over a whole range of stress intensities was performed. In Figure 7, the fatigue growth rate, da/dN, for six averaged curves of PET*96 h and PET control sample versus the applied stress intensity factor ΔK at the crack tip was plotted to investigate the FCP behavior.

In general, da/dN curves can be subdivided into three different crack propagation regions, as shown in Figure 7. Region I describes the crack propagation after reaching the threshold value ∆K_th_. Below this value, crack propagation is negligibly low. Region II represents stable crack growth according to the Paris–Erdogan Equation da/dN = C (∆K)^m^, where C and m are material constants. Region III is determined by instable crack propagation until the final fracture of the sample. 

In the case of PET, the behavior of the material did not significantly differ from the control sample in region I, except for one enzyme-incubated sample with a lower threshold value. This can be explained by the inhomogeneities of biotic treatment, as observed in SEM (Figure 3). However, with increasing mechanical load upon region II, the empowered crack propagation rates of the PET*96 h samples indicate the reduction in crack growth resistance. These results are consistent with the formation of BHET species on the surface, which is associated with the breakdown of individual polymer chains and material deterioration. The degradation of individual polymer chains results in a decreasing number of links between the crystalline PET units, so-called tie-molecules. With decreasing tie-molecule density, the craze network is destabilized, and the induced dynamic load causes molecular fracture, resulting in macroscopically brittle behavior [46,47]. Additionally, the development of a craze network could be interrupted by those additional crystalline units of BHET oligomers. Consequently, complete failure of the enzyme-treated material can be investigated at lower stress intensities compared to the control samples in region III accompanied by a faster crack growth velocity. 

**Figure 8:**
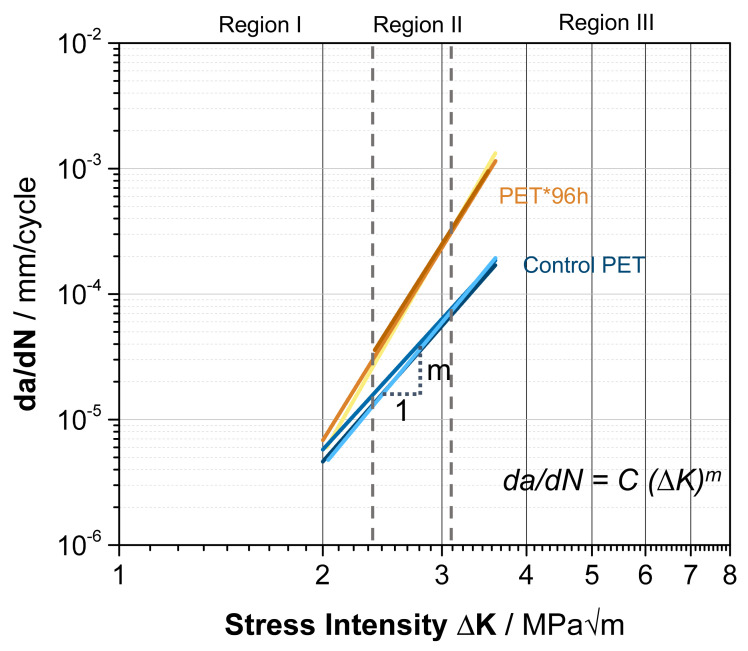
Power function fit of the FCP data on the linear range in region II in a double logarithmic scale with curves for the PET control (light blue, blue, dark blue) and PET*96 h (yellow, orange, dark orange). The averaged parameters for the fit are m_b_ = 5.5 ± 1.1, C_b_ = 10–7.9 ± 0.5 for the PET control samples, and m_e_ = 10.2 ± 1.4, C_e_ = 10–8.4 ± 0.6 for PET*96 h.

To quantify the influence of enzymatic degradation, a fit was applied to each measurement in the linear-dependent stable crack propagation in region II (Figure 8). The linear fit in a logarithmic scale shows the impact of enzymes in combination with mechanical stress. Without enzymatic treatment, an average slope of m_b_ = 5.5 ± 1.1 can be calculated according to y = a × x^m^ for the PET control. In comparison to the PET*96 h sample, the value of the slope increased to m_e_ = 10.2 ± 1.4. This clearly identifies an impact of PETase on the degradation behavior on the path from macro- to microplastic by fragmentation due to mechanical stress.

## 4. Conclusions

The time-dependent investigation of PET samples exposed to PETase showed the formation of colloidal structures growing with incubation time and thereby increasing the surface roughness. These structures could be attributed to BHET dimer affecting the crystalline structure on the PET surface. The interruption of the crystalline order accompanied by the degradation of single polymer chains facilitated the crack propagation under mechanical stress, resulting in earlier failure of the incubated sample compared to the control. Within this study, we showed that biotic factors have a relevant impact on the pathway from macro- to microplastic, as material properties play a decisive role in the progress of plastic fragmentation. The investigations further hinted at certain preferences in substrate binding to PETase. Our results provide a first step towards understanding the impact of enzymatic treatment through the establishment of a reliable method for the quantification and development of a new experimental setup. The applied methods provide reliable data for the quantitative analysis of biotic impact by enzymatic degradation.

The results gained from this pilot study provide the basis for future interdisciplinary research combining biochemistry and material science. Here, we established a method for the determination of enzymatic activity on material properties in a quantifiable fashion. This protocol can act as a foundation towards a full understanding of the interplay between enzyme and material. Future experiments must consider time-resolved analysis as well as a detailed characterization of the surface erosion and chemistry. Moreover, to fully cover the degradation process in nature with its implications on material properties, there is a need to include abiotic factors such as temperature, pH, stage of weathering of PET material, and material composition with the parameters of biotic degradation like enzyme type or concentrations, incubation time, and buffer composition. The described method now provides a reliable platform to perform these studies in the future.

## Data Availability

The data set used in this study is published on Zenodo, https://doi.org/10.5281/zenodo.5657200, accessed on 10 November 2021.
